# Heart rhythm-synchronized fibrin flap in a glaucoma tube shunt: The heartbeat acts as a drainage pump for the aqueous humor: A case report

**DOI:** 10.1097/MD.0000000000026603

**Published:** 2021-07-09

**Authors:** Hirotaka Tanabe, Shunsuke Nakakura, Hitoshi Tabuchi

**Affiliations:** a Department of Ophthalmology, Tsukazaki Hospital, Himeji, Hyogo; b Department of Technology and Design Thinking for Medicine, Hiroshima University Graduate School of Biomedical and Health Sciences, Hiroshima, Japan.

**Keywords:** aqueous humor, Baerveldt glaucoma tube, fibrin, glaucoma, heartbeat

## Abstract

**Rationale:**

The amount of aqueous humor that is constantly produced by the ciliary processes and the equal amount that flows out through the trabecular meshwork via the drainage angle or through the uveoscleral route is very small (2–3 μg/min each), representing approximately 1% of the content of the anterior chamber; therefore, it is challenging to visualize its flow.

**Patient concerns:**

A 69-year-old man who had high intraocular pressure (IOP) (>20 mm Hg) with the maximum glaucoma eyedrop dose and presented with severe visual field loss (Humphrey Field Analyzer 30–2: –26.32 dB) had been implanted with a 350-mm^2^ Baerveldt tube of the aqueous chamber type for refractory open-angle glaucoma. The IOP ultimately decreased (<15 mm Hg) with no need for glaucoma eyedrops.

**Diagnoses:**

After the procedures, a fibrin membrane repeatedly formed on the anterior surface of the intraocular lens.

**Interventions:**

This issue was resolved by two rounds of neodymium-doped yttrium aluminum granet (Nd:YAG) laser surgery and prescription steroidal eyedrops.

**Outcomes:**

During the laser surgery, an unusual and unintended fibrin flap appeared at the opening of the Baerveldt tube; this flap moved synchronously with the heartbeat, as verified by checking the pulse at the radial artery of the wrist. The fibrin flap mimicked the behavior of a cardiac valve, and the aqueous humor and stray fibrin particles mimicked the blood in the chambers of the heart. Although the Baerveldt tube itself is an artificial instrument that is not present in normal human eyes, we hypothesize that our observation shows the fundamental mechanism of aqueous humor drainage.

**Lessons:**

This novel, vividly descriptive observation highlights the important role of the heartbeat as a drainage pump in aqueous humor flow dynamics and IOP homeostasis, which are treatment targets for glaucoma, the leading cause of blindness.

## 1 Introduction

The amount of aqueous humor that is constantly produced by the ciliary processes and the equal amount that flows out through the trabecular meshwork via the drainage angle or through the uveoscleral route is very small (2–3 μg/min each), representing approximately 1% of the content of the anterior chamber; therefore, it is challenging to visualize its flow. Goldmann^[1]^ noted that the pulsatile flow of aqueous humor into the aqueous veins is cyclic and synchronous with the ocular pulse waves that originate in Schlemm canal (SC). Additionally, Phillips et al^[2]^ hypothesized that the ocular pulse waves are induced by the change in the choroidal vascular volume as the cardiac pulse oscillates between diastole and systole, acting as a piston for an aqueous pump. Johnstone^[3]^ observed evidence suggesting pulsatile aqueous flow into the collector channel and SC in Stegmann videotapes of a gonioscopic view of the anterior chamber angle, in which the episcleral veins were compressed with the flange of a goniolens to cause slight blood reflux into the collector channels; the aqueous humor in the collector channels is tinged with blood, which renders its flow visible. Johnstone^[3]^ explained that the ocular pulse can induce pulsatile motion of the trabecular meshwork outward into SC, causing a decrease in total volume in the lumen of SC and a transient increase in the pressure within this canal, which allows the intraocular pressure (IOP) increase to elicit a pulse wave expelling aqueous humor from SC. Xin et al^[4]^ recently supported this idea by showing the pulse-dependent motion of the trabecular meshwork using phase-sensitive optical coherence tomography. In this report, we would like to present a case in which important roles of the heartbeat on the aqueous humor dynamics are suggested.

## 2 Case report

**Figure 1:**
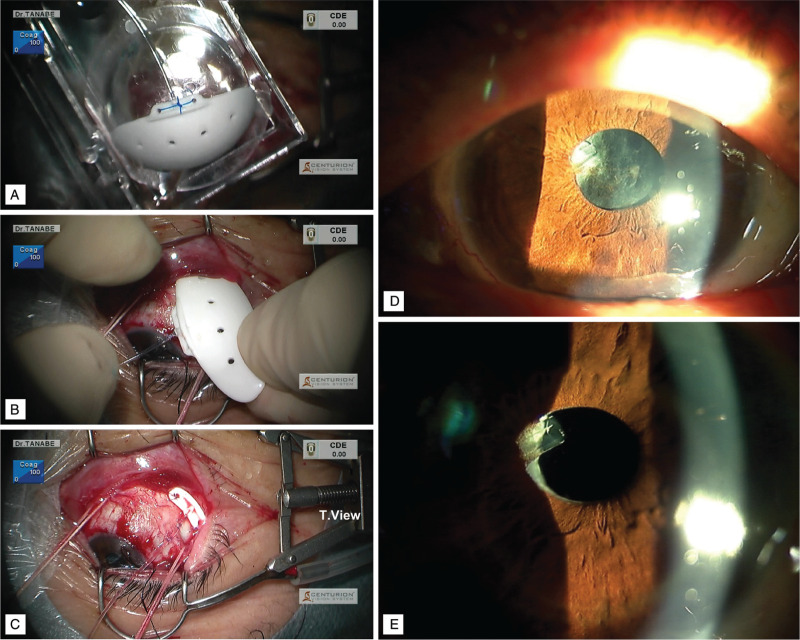
Baerveldt tube implantation and postoperative fibrin membrane formation on the intraocular lens. (A) The 350-mm^2^ Baerveldt tube (aqueous chamber type). (B) Baerveldt tube implantation. (C) Surgical field during the tube-shunt surgery. The eye is held open with the Tanabe Temporal View Speculum (T. View, TTVS) (Eye Technology [UK] and M.E. Technica [Japan]) to achieve a maximum view of the temporal surgical field. (D) Slit-lamp microscopy shows a fibrin membrane on the intraocular lens. (E) The fibrin membrane was successfully removed by Nd:YAG laser surgery. Nd: YAG = neodymium-doped yttrium aluminum garnet.

**Figure 2:**
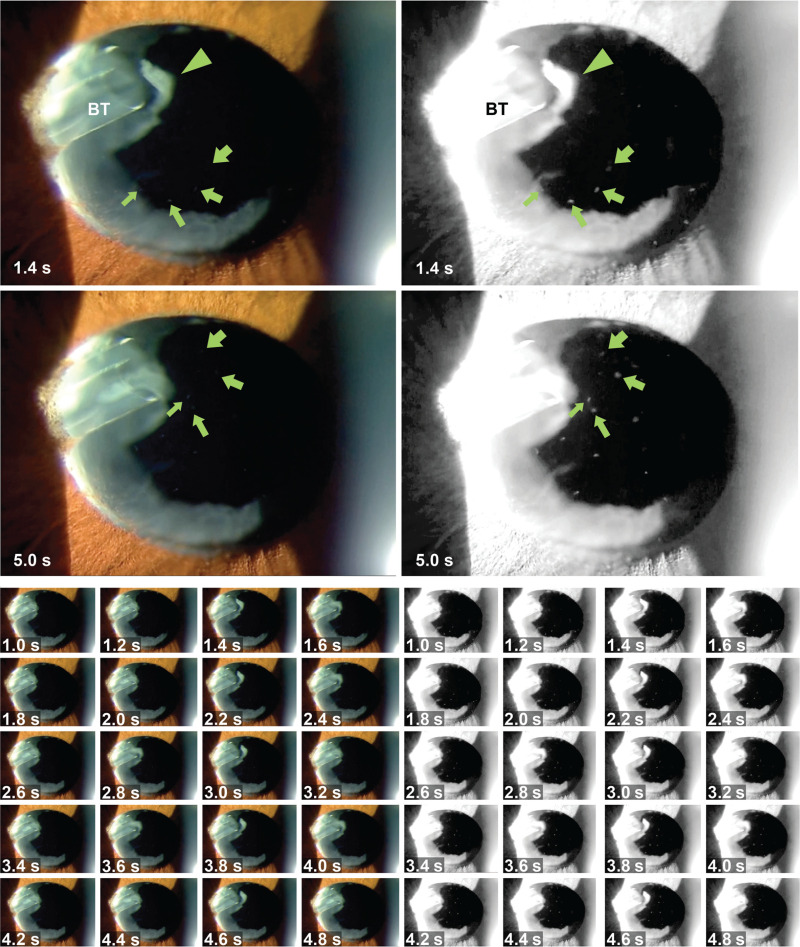
Heart rhythm-synchronized fibrin flap on the glaucoma tube shunt. Twenty serial photographs (taken across 4 s from 1 to 5 s; see Videos S2 and S3, Supplemental Digital Contents 2 and 3, which demonstrate a heart rhythm-synchronized fibrin flap in a glaucoma tube shunt) show the fibrin flap (arrowhead), which moved synchronously with the heartbeat (0.8 s/pulse). Representative photographs demonstrate that the aqueous humor along with stray fibrin particles (arrows) were being drained in synchrony with the pulse. BT = Baerveldt tube.

A 69-year-old man was referred to our hospital for glaucoma surgery. His right eye had high IOP (>20 mm Hg) with the maximum glaucoma eyedrop dose and presented severe visual field loss (Humphrey Field Analyzer 30–2: –26.32 dB). Cataract surgery, suture trabeculotomy ab interno, trabeculectomy with ExPRESS implantation, bleb revision, and tube-shunt surgery (350-mm^2^ Baerveldt, aqueous chamber type) (Fig. 1A–C) were performed sequentially for refractory open-angle glaucoma. The IOP ultimately decreased (<15 mmHg) with no need for glaucoma eyedrops. After the procedures, a fibrin membrane repeatedly formed on the anterior surface of the intraocular lens; this issue was resolved by 2 rounds of neodymium-doped yttrium aluminum garnet laser surgery and prescription steroidal eyedrops (Fig. 1D and E). During laser surgery, an unusual and unintended fibrin flap appeared at the opening of the Baerveldt tube; this flap moved synchronously with the heartbeat, as verified by checking the pulse at the radial artery of the wrist (Fig. 2; see Videos S1–3, Supplemental Digital Contents 1–3, which demonstrate a heart rhythm-synchronized fibrin flap in a glaucoma tube shunt). The aqueous humor, along with stray fibrin particles, drained in synchrony with the pulse, corroborating a previous speculation about the dynamics of the aqueous humor.

## 3 Discussion

In our observation of the patient with the Baerveldt tube, the fibrin flap mimicked the behavior of a cardiac valve, and the aqueous humor with stray fibrin particles mimicked the blood in the chambers of the heart. Although the Baerveldt tube itself is an artificial instrument that is not present in normal human eyes, we hypothesize that our observation shows the fundamental mechanism of aqueous humor drainage. Adequate aerobic exercise is known to lower IOP and glaucoma risk, although the mechanisms, which may include decreased norepinephrine levels, increased colloid osmotic pressure, hypocapnia, increased blood lactate, and a link with body weight, are complicated and not fully understood.^[5]^ Regulation of fluid movement in the eye observed in this case may partly explain why exercise is helpful in preventing glaucoma.

## 4 Conclusion

This novel, vividly descriptive observation, in the context of the previous representative studies discussed here, suggests the important role of the heartbeat as a drainage pump in aqueous humor flow dynamics and IOP homeostasis, which are treatment targets for glaucoma, the leading cause of blindness (see Video S4, Supplemental Digital Content 4, which is a video abstract).

## Acknowledgments

The authors thank all of the staff of Tsukazaki Hospital who were involved in this patient's care.

## Author contributions

**Conceptualization:** Hirotaka Tanabe.

**Data curation:** Hirotaka Tanabe.

**Investigation:** Hirotaka Tanabe.

**Methodology:** Hirotaka Tanabe.

**Project administration:** Hirotaka Tanabe.

**Resources:** Hirotaka Tanabe, Hitoshi Tabuchi.

**Supervision:** Hirotaka Tanabe.

**Validation:** Hirotaka Tanabe, Shunsuke Nakakura.

**Visualization:** Hirotaka Tanabe.

**Writing – original draft:** Hirotaka Tanabe.

**Writing – review & editing:** Hirotaka Tanabe.

## Supplementary Material

Supplemental Digital Content

## Supplementary Material

Supplemental Digital Content

## Supplementary Material

Supplemental Digital Content

## Supplementary Material

Supplemental Digital Content
